# Integrating climate change adaptation into hypertension care: an evidence based framework for climate resilient primary healthcare and public health planning in Limpopo Province, South Africa

**DOI:** 10.3389/fpubh.2026.1937901

**Published:** 2026-10-29

**Authors:** Salphy Mamoropo Mmusi, Mamare Adelaide Bopape, Tebogo Maria Mothiba

**Affiliations:** 1 Department of Nursing Science, University of Limpopo, Polokwane, South Africa; 2 Faculty of Health Science, Executive Dean’s Office, University of Limpopo, Polokwane, South Africa; 3 National Research Foundation, SARChi Chairs, University of Limpopo, Polokwane, South Africa

**Keywords:** climate change adaptation, climate resilient healthcare, health systems strengthening, hypertension management, primary healthcare

## Abstract

**Background:**

Climate change poses a growing public health challenge, particularly for individuals living with chronic conditions such as hypertension. Climate related stressors, including extreme heat and cold, may adversely affect blood pressure control and self-management, highlighting the need for context specific adaptation strategies.

**Purpose:**

This study aimed to develop evidence based coping strategies to support hypertensive patients in adapting to climate change and managing the health effects of changing weather conditions, contributing to Sustainable Development Goal (SDG) 3 (Good Health and Well-being) and SDG 13 (Climate Action).

**Methods:**

The strategies were developed from the empirical findings of a mixed-methods study guided by Roy’s Adaptation Model and Thompson’s Five Stage Strategy Development Framework. Data were collected from 244 hypertensive patients using self-developed questionnaires and from 15 patients through semi structured interviews in selected districts of Limpopo Province, South Africa. Quantitative data were analysed using SPSS version 28, while qualitative data were analysed using Tesch’s eight step method. Findings were integrated using a joint display to identify areas of convergence, divergence, and expansion. The resulting strategies were subsequently reviewed through one round of Modified Delphi consultation to establish expert consensus regarding their clarity, relevance, feasibility, practicality, and applicability.

**Findings:**

The study identified limited awareness of climate change and its health impacts, challenges in hypertension management during extreme temperatures, inconsistent coping practices, and a lack of structured adaptation guidance. These findings informed the development of seven evidence-based strategies addressing climate informed healthcare, self-management during extreme weather, behavioural and lifestyle modification, information and coping, patient education and empowerment, climate resilient lifestyle practices, and emergency preparedness. Collectively, the strategies translate patient experiences into a structured approach for strengthening adaptive capacity, resilience, and climate responsive hypertension management.

**Implications:**

The developed framework provides a practical approach for integrating climate adaptation into hypertension management and strengthening climate-resilient primary healthcare. It offers a patient-centred model that can inform health service planning, community engagement, policy development, and chronic disease programmes while supporting progress towards SDGs 3 and 13.

## 1 Introduction

Climate change is increasingly recognised as one of the most significant global public health challenges of the 21st century, with profound implications for the prevention, management, and outcomes of non communicable diseases (NCDs), including hypertension (1). Climate related hazards such as rising temperatures, extreme heat and cold events, changing rainfall patterns, droughts, floods, and air pollution contribute to increased cardiovascular stress and have been associated with elevated morbidity and mortality among vulnerable populations (2). Hypertensive patients are particularly susceptible to these environmental stressors because climate variability may influence physiological responses, medication effectiveness, health seeking behaviours, access to healthcare services, and the ability to maintain effective self management practices (3). Consequently, climate change presents an emerging challenge for achieving sustainable hypertension control and reducing cardiovascular disease burden globally.

The intersection between climate change and hypertension is of particular concern in low and middle income countries, where health systems already face substantial challenges in managing the growing burden of NCDs. In Africa, hypertension prevalence has increased substantially over recent decades, with many countries experiencing difficulties related to early detection, treatment continuity, medication adherence, and long term disease management (4). These challenges are further intensified by poverty, limited healthcare infrastructure, inadequate access to essential medicines, and climate related disruptions affecting health service delivery (5). Studies from African countries have highlighted that extreme weather events, including heatwaves, droughts, and floods, can worsen health vulnerabilities by disrupting healthcare access, reducing food and water security, and increasing exposure to environmental risks among individuals living with chronic illnesses (6).

South Africa faces a dual burden of climate vulnerability and a high prevalence of hypertension. Although the country has made progress in strengthening NCDs prevention and management through primary healthcare initiatives, persistent challenges remain, particularly in rural and underserved communities (7). Rural provinces such as Limpopo are disproportionately affected due to geographical barriers, limited healthcare resources, socio economic inequalities, and increased exposure to climate variability (8). Patients in these settings may experience difficulties accessing healthcare facilities during extreme weather events, maintaining medication supplies, obtaining health information, and implementing appropriate lifestyle modifications. Furthermore, limited integration of climate change considerations into routine hypertension care may reduce the capacity of healthcare providers and patients to anticipate and respond effectively to climate related health risks (9).

Although the relationship between climate change and cardiovascular health is increasingly recognised, existing evidence has primarily focused on describing climate related health impacts rather than developing practical adaptation strategies for individuals living with hypertension. There remains limited evidence on context specific, patient centred coping strategies that enable hypertensive patients to adapt to changing environmental conditions, particularly within primary healthcare settings in South Africa and other African contexts (10). Current hypertension management approaches rarely incorporate climate related risks, climate literacy, early warning mechanisms, or tailored patient education to support adaptation. This knowledge gap highlights the need for integrated, climate responsive approaches that strengthen healthcare systems while empowering patients to maintain effective self-management and reduce climate related cardiovascular risks (9).

Beyond addressing hypertension, this gap reflects a broader challenge within public health systems, namely the limited integration of climate adaptation into routine chronic disease management. As climate related health risks increasingly affect vulnerable populations living with NCDs, there is a need for practical frameworks that support patient centred care while strengthening health system preparedness, cross-sectoral collaboration, and community resilience. Developing context specific adaptation strategies therefore has the potential to inform climate resilient approaches applicable across chronic disease programmes and primary healthcare services, particularly in resource constrained settings.

Therefore, this study aimed to develop climate change adaptation coping strategies for hypertensive patients, contributing to SDG 3 and 13. Specifically, the study sought to strengthen healthcare workers’ capacity to provide climate responsive hypertension care, enhance patient education and self-management, improve healthcare delivery systems through climate informed approaches, promote early warning and preparedness mechanisms, and foster community engagement and partnerships. Grounded in RAM, the resulting framework translates patients’ lived experiences into evidence informed strategies that extend beyond individual coping by supporting health system planning, coordinated stakeholder action, and climate resilient primary healthcare. Although developed within the context of hypertension, the framework provides a transferable process for integrating climate adaptation into chronic disease management and broader public health planning in climate vulnerable settings such as Limpopo Province, South Africa.

## 2 Data sources and methods

The study employed a mixed-methods convergent parallel design involving the concurrent collection and analysis of quantitative and qualitative data, followed by their integration to compare, corroborate, and expand the findings and provide a comprehensive understanding of the research topic. The quantitative component comprised 244 completed self-developed questionnaires administered to hypertensive patients in selected districts of Limpopo Province. Systematic sampling was used to select eligible patients from selected district hospitals. The target population comprised 400 patients who had received hypertension treatment for at least 3 years, from which a sample of 244 was determined using Slovin’s formula, with a 5% margin of error and a 95% confidence level. The qualitative component comprised 15 semi-structured interviews with hypertensive patients selected using homogeneous purposive sampling. Recruitment continued until data saturation was reached, with no new codes or themes emerging from subsequent interviews. Quantitative data were analysed using the Statistical Package for the Social Sciences (SPSS), Version 28, while qualitative data were analysed using Tesch’s eight-step method of data analysis. The quantitative and qualitative findings were integrated to identify areas of convergence, complementarity, and divergence, which informed the development of draft climate change adaptation strategies. The draft strategies were subsequently developed and structured using Roy’s Adaptation Model (RAM) and Thompson et al.’s Strategy Development Framework, as illustrated in Figure 1.

**Figure 1:**
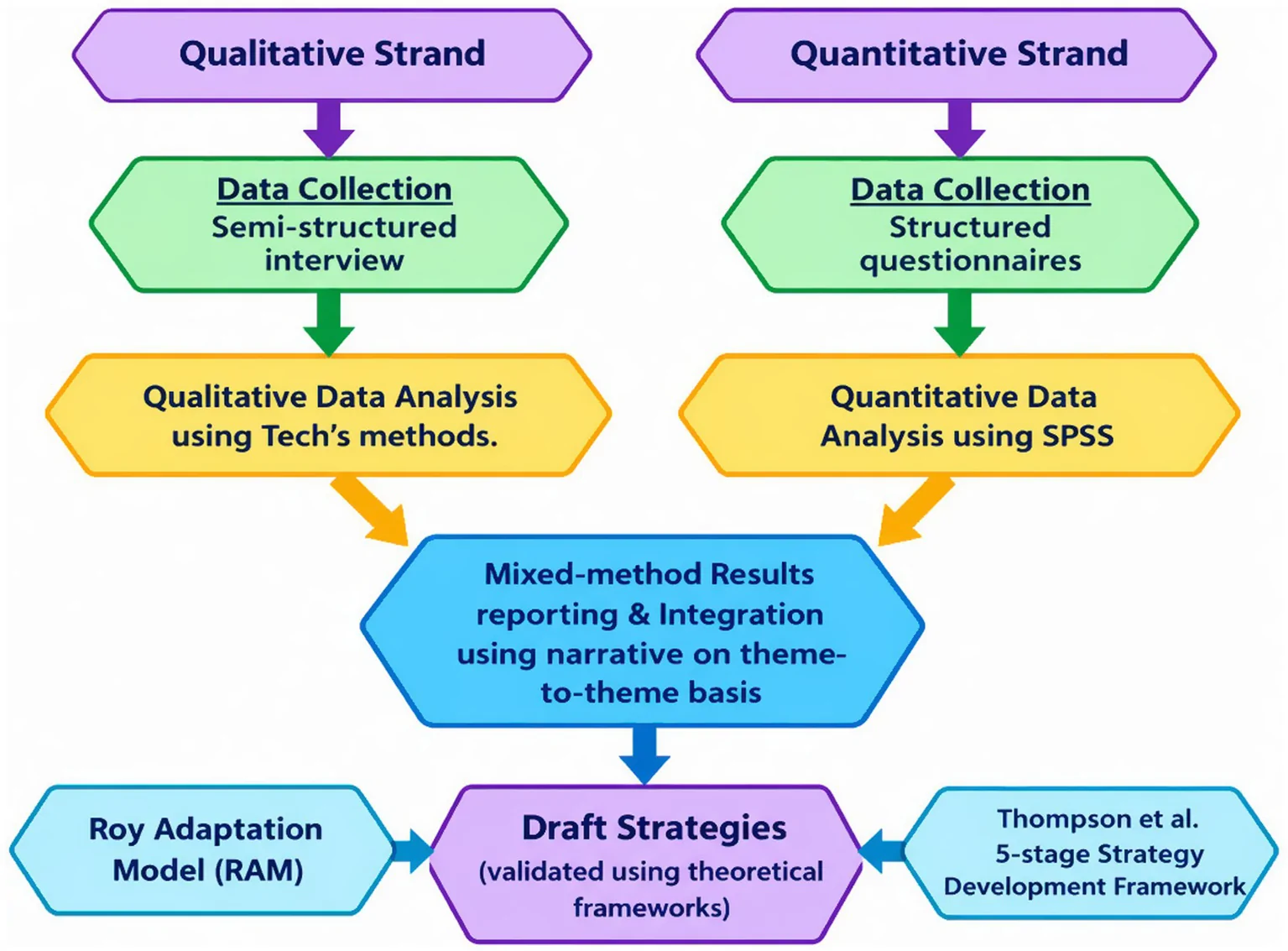
Flowchart of the mixed methods convergent parallel design showing the integration of empirical findings with Roy’s Adaptation Model and Thompson et al.’s Strategy Development Framework for developing draft climate change adaptation strategies (54).

### 2.1 Integration of quantitative and qualitative findings and implications for strategy development

The merged qualitative and quantitative findings demonstrated strong convergence in hypertensive patients’ recognition and experience of climate change, particularly extreme heat and cold. Extreme heat was most prominent, with 84% reporting very hot summer temperatures and 65.2% noticing changes in blood pressure during extreme heat. Divergence was identified in patients’ knowledge of adaptation strategies, with 58% unaware of appropriate cooling techniques. The qualitative findings further expanded the quantitative results by illustrating practical coping strategies, including increased water intake, dietary changes, consumption of traditional foods, and use of indigenous remedies. These findings highlight the need for targeted information, education, and practical guidance to strengthen patients’ capacity to manage hypertension during extreme weather events. The integrated findings therefore provided an empirical basis for the development and refinement of patient centred climate change adaptation strategies aimed at promoting effective hypertension management and resilience.

## 3 Development of strategies

The development of strategies was guided by the stages of strategy development (11) (Figure 2).

**Figure 2:**
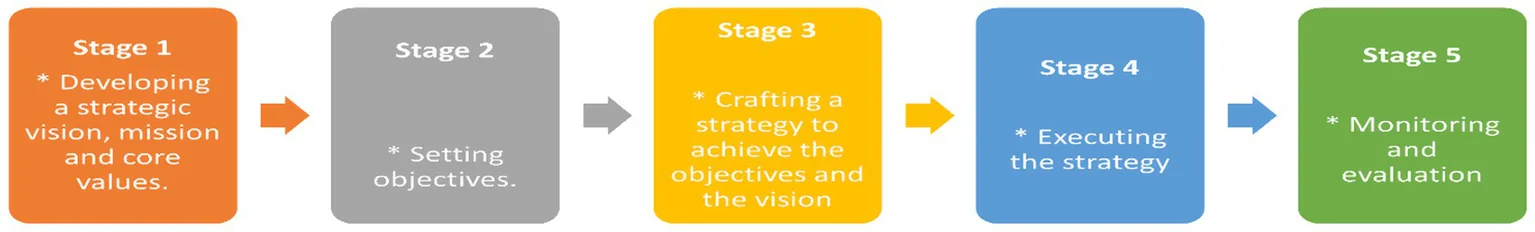
Stages of strategy development [Thompson et al. (12), p. 19].

This study developed climate adaptation strategies to enhance the capacity of hypertensive patients to respond to climate related health challenges. The strategies were designed to support patients in managing the effects of changing environmental conditions and strengthening resilience among vulnerable populations in Limpopo Province, South Africa. These strategies contribute to SDG 3 by promoting improved health outcomes and sustainable hypertension management, while also supporting SDG 13 through the promotion of climate adaptation and resilience.

Beyond individual coping behaviours, the developed climate adaptation framework provides a structured approach for integrating climate change adaptation into hypertension management and advancing a shift from fragmented, disease specific interventions towards systemic and cross sectoral public health planning. Guided by Thompson’s Five Stage Strategy Development Framework and informed by RAM, the framework links patient level adaptation with healthcare system strengthening, policy development, and multisectoral collaboration. It recognises that climate related health risks among hypertensive patients are shaped by interconnected environmental, social, economic, and healthcare factors that require coordinated responses beyond the health sector alone (11).

### 3.1 Stage 1: developing strategic vision, mission, and core values

Stage 1 established the vision, mission, and core values of the climate change adaptation strategy, informed by the merged qualitative and quantitative findings. The findings showed that hypertensive patients recognised and experienced climate related changes, particularly extreme heat and cold, while gaps in knowledge and management practices highlighted the need for targeted education and practical guidance. These findings informed a vision of an informed, supported, and resilient hypertensive population and a mission to strengthen healthcare systems in delivering climate responsive care. The core values of climate responsiveness, equity, empowerment, quality care, and collaboration provided guiding principles for implementation and stakeholder engagement. Collectively, these elements translated the study findings into a strategic direction for developing patient-centred climate change adaptation strategies to strengthen hypertension management and resilience during extreme weather events.

#### 3.1.1 Vision

A population of hypertensive patients that is informed, supported, and resilient in adapting to climate change related health challenges.

#### 3.1.2 Mission

To strengthen healthcare units in Limpopo Province to deliver high quality, climate responsive care to patients living with hypertension.

#### 3.1.3 Core values

- Patient-centred care: Prioritising the dignity, preferences, and needs of patients living with hypertension in all aspects of care delivery.
- Climate responsiveness: Integrating climate change related risks into healthcare planning, service delivery, and disease management approaches.
- Quality and excellence: Ensuring the provision of safe, effective, and evidence informed healthcare services.
- Empowerment and education: Enhancing patient and healthcare provider knowledge to promote self-management and informed decision making.
- Equity and accessibility: Ensuring fair and equitable access to healthcare services for all patients.
- Collaboration and partnership: Strengthening teamwork and partnerships among healthcare professionals, communities, and relevant stakeholders.

### 3.2 Stage 2: setting objectives

The strategic objectives translated the merged qualitative and quantitative findings into measurable, coordinated priorities for climate-resilient hypertension care. The findings showed that hypertensive patients experience extreme heat and cold, while gaps in knowledge and management practices highlighted the need for targeted education and practical guidance. These findings, including patient identified coping practices, informed the following five strategic objectives, which provided a systematic framework for developing the climate change adaptation strategy:

1. Strengthen clinical capacity.

Enhance healthcare workers’ capacity to effectively manage hypertension within the context of climate related stressors and environmental changes.

1. Enhance patient education and awareness.

Improve patients’ knowledge regarding hypertension self-management, treatment adherence, and the health risks associated with climate variability and extreme weather conditions.

1. Improve service delivery systems.

Integrate climate responsive and adaptive approaches into primary healthcare services to strengthen continuity, accessibility, and quality of care for hypertensive patients.

1. Promote early warning and preparedness systems.

Support the development and implementation of timely early warning mechanisms and preparedness strategies to enable effective responses to climate extremes affecting patients with hypertension.

1. Strengthen community engagement and partnerships.

Foster collaboration among healthcare facilities, communities, and key stakeholders in Limpopo Province to support sustainable, community based responses to hypertension and climate related health challenges.

Collectively, these objectives extend hypertension management beyond healthcare facilities by addressing the social, environmental, and structural factors that influence patients’ ability to adapt to climate related health risks and support sustainable, patient-centred climate adaptation in Limpopo Province.

### 3.3 Stage 3: crafting strategies to achieve the mission, vision, and objectives

Stage 3 operationalised the merged qualitative and quantitative findings by translating the identified experiences, knowledge gaps, and coping practices of hypertensive patients into evidence-informed, patient centred strategies. The findings demonstrated patients’ recognition and experience of climate related changes, knowledge and management practices, highlighted the need for targeted education and practical guidance. The qualitative findings further expanded the quantitative results by identifying patients’ existing coping practices. These findings provided the empirical basis for developing strategies that strengthen patients’ capacity to adapt to climate related health risks.

The strategies operationalise the study’s vision, mission, and strategic objectives through coordinated action across individual, community, healthcare system, and policy levels. They incorporate the perspectives and roles of patients, healthcare providers, and relevant stakeholders, including nurses, physicians, home based carers, public health practitioners, policymakers, environmental and meteorological services, disaster management structures, and communities. Guided by RAM, hypertensive patients are recognised as active participants in the adaptation process, supported by responsive healthcare services, social networks, and community resources. By defining stakeholder roles and aligning responsibilities with available resources and healthcare priorities, Stage 3 provides a practical mechanism for translating the integrated findings into coordinated climate-resilient hypertension care.

#### 3.3.1 Goal of the strategy

The overall goal of these coping strategies was to enable hypertensive patients to effectively manage their condition and mitigate additional risks associated with climate change. Once implemented, these strategies will provide guidance to the Department of Health in strengthening the adaptation of hypertensive patients to climate related health challenges. Furthermore, the developed strategies will support policymakers in the development and implementation of climate change and health-related policies and interventions. They will also enhance the knowledge and capacity of healthcare providers and home based carers to provide appropriate information and support to hypertensive patients regarding climate change adaptation.

#### 3.3.2 Principles of the strategy

The coping strategies for hypertensive patients’ adaptation to climate change were developed based on patient rights-based standards for health and wellbeing information and services. According to Downs et al., ensuring that health information and services meet human rights standards requires attention to specific quality of care components, including functional health systems (facility readiness) and healthcare providers’ technical competency (process of care) (12).

A functional health system requires appropriate policies and protocols, adequate physical infrastructure, relevant training curricula, supportive supervision, essential equipment and supplies, information systems, and mechanisms for receiving and responding to patient feedback. These components are essential for ensuring the delivery of quality, accessible, and climate responsive hypertension care (13).

Technically competent healthcare providers: During the care process, healthcare providers should possess the necessary technical competencies, including effective interpersonal communication, counselling skills, and the ability to provide relevant health education. These competencies should support the provision of information related to patient education and awareness, environmental risk management, hydration and nutrition, medication management, access to healthcare services, community support and resources, mental health and stress management, policy advocacy, and research. By integrating these competencies, healthcare providers can assist hypertensive patients in effectively managing their condition amid climate related challenges, thereby improving health outcomes and quality of life (14).

#### 3.3.3 Target audience

The target audience for the strategy includes key stakeholders involved in hypertension management and climate adaptation within the healthcare system. These include nurses working in hospital outpatient departments, healthcare centres, and clinics; physicians and general practitioners providing care within these settings; and other healthcare workers involved in patient management.

Home based carers responsible for conducting household visits and nurses involved in school health programmes also form part of the target audience. At the health system level, implementation will be supported by the Provincial Quality Team in Limpopo Province and policymakers within the Limpopo Department of Health (55).

### 3.4 Stage 4: final strategies

This stage involved refining and finalising the developed strategies based on findings from the quantitative and qualitative phases of the study. The process consolidated the evidence informed interventions into seven strategic areas that integrate individual, community, healthcare system, and policy level responses. For example, climate informed healthcare service strategies require health facilities to incorporate climate related risks into planning and service delivery, while emergency preparedness strategies require collaboration among healthcare services, disaster management structures, and local authorities. Similarly, patient education, climate adaptation information, coping strategies, and climate resilient lifestyle interventions require coordinated partnerships between healthcare providers, community structures, and communication platforms. Collectively, these strategies establish pathways for shared accountability, resource mobilisation, and coordinated implementation across sectors. The final strategies were designed to address identified gaps and challenges while strengthening existing approaches to support hypertensive patients’ adaptation to changing weather conditions and climate related health risks (11) (Table 1).

**Table 1:** Biographic profile of experts participating in the modified Delphi process.

Expert ID	Age	Gender	Qualification	Professional role	Experience (Years)	Areas of expertise
Expert 1	75	M	PhD in Medicine	HOD	53	Family Medicine
Expert 2	48	F	MMED, BCUR, Student(PhD in Medicine)	Family Physician	27	Family Medicine
Expert 3	36	F	PhD In Nursing, Nursing Education	Clinical Lecture	16	Nursing Research
Expert 4	54	M	PhD In Public Health	Research Lecturer	32	Public Health Research
Expert 5	56	F	Nursing Specialist (PHC)	Operational manager	36	Community Nursing
Expert 6	48	F	Diploma in Nursing Science	Professional Nurse	29	Community Nursing

The final strategies include:

1. Strategy 1: Implementation of climate informed healthcare service measures
2. Strategy 2: Selfcare management during extreme heat conditions
3. Strategy 3: Behavioural and lifestyle interventions
4. Strategy 4: Information coping strategies
5. Strategy 5: Patient education and empowerment
6. Strategy 6: Climate resilient lifestyle education strategies
7. Strategy 7: Emergency preparedness strategies

#### 3.4.1 Validation of strategies

The developed strategies were validated using a Modified Delphi Technique to assess their clarity, relevance, feasibility, practicality, applicability, and alignment with the study objectives, vision, and mission. Six experts were purposively selected based on their professional expertise and extensive experience in hypertension management, healthcare practice, and research. The expert panel comprised two physicians, two professional nurses, and two academics, with all experts having more than 15 years of professional experience, as presented in Table 2. The diversity of the expert panel enabled the proposed strategies to be assessed from clinical, nursing, academic, and public health perspectives.

**Table 2:** Modified Delphi results and expert recommendations for refinement of the draft strategies.

Delphi validation result	Finding
Number of experts	6
Number of criteria	24
Total expert-item ratings	144
“Yes” responses	143 (93.3%)
“No” responses	1 (0.7%)
Criteria with 100% agreement	23 (95.8%)
Criterion with 83.3% agreement	1 (4.2%)
Consensus threshold	≥80%
Criteria achieving consensus	24/24 (100%)

The developed strategies underwent a one round Modified Delphi process involving six purposively selected experts. An electronically administered questionnaire comprising 24 criteria across four domains Goals and Practice, Context of the Coping Strategies, Explanation of the Coping Strategies, and Evidence to Support the Coping Strategies was used to assess the clarity, relevance, feasibility, practicality, applicability, and alignment of the draft strategies with the study objectives. Each expert independently rated all criteria using a two-point scale (1 = Yes; 2 = No) and was provided with an opportunity to provide qualitative comments, yielding 144 expert-item ratings.

Overall, 143/144 ratings (93.3%) were affirmative, with one negative response (0.7%). Twenty three criteria achieved 100% agreement, while one achieved 83.3% agreement; consequently, all 24 criteria met the predefined 80% consensus threshold. Qualitative feedback obtained during the Modified Delphi round subsequently informed refinement of the draft strategies. Experts recommended strengthening the description and implementation guidance for Strategy 5, incorporating supporting evidence into the Patient Education and Empowerment Strategy, explicitly applying adult education principles, and strengthening consideration of rural resource constraints, health literacy, behaviour and lifestyle change, success indicators, and potential conceptual overlap between Strategies 1 and 4. These recommendations were incorporated into the subsequent refinement of the strategies. Because the revised strategies were not returned to the experts for a second round of validation, the reported consensus applies to the draft strategies assessed during the Modified Delphi round and should not be interpreted as consensus on the subsequently refined version.

The draft strategies provide a contextually responsive approach to climate sensitive hypertension management in primary healthcare settings. Their implementation may be supported through monitoring indicators addressing patient adaptation, healthcare provider competencies, climate health surveillance, service readiness, community engagement, and policy implementation. By linking patient centred adaptation with health system preparedness, community participation, and multisectoral collaboration, the framework provides a basis for strengthening climate-resilient hypertension management in resource-constrained settings and contributes to the objectives of SDG 3 and SDG 13.

### 3.5 Stage 5: monitoring and evaluation of strategies

Monitoring and evaluation will be undertaken to determine whether the climate change adaptation strategies are implemented as intended and whether they achieve the desired outcomes among hypertensive patients in primary healthcare settings. Implementation monitoring will assess fidelity, reach, acceptability, feasibility, and uptake of the strategies using routine records, implementation checklists, attendance registers, healthcare provider reports, and patient feedback.

Effectiveness evaluation will assess changes in patients’ knowledge of climate-related health risks, self-management practices, preparedness for extreme weather events, coping strategies, medication adherence, blood pressure monitoring, and relevant hypertension related outcomes. Quantitative outcome measures will be complemented by qualitative interviews with patients and healthcare providers to explore perceived benefits, barriers, facilitators, and contextual factors influencing implementation. Monitoring and evaluation findings will be used to identify gaps, guide continuous refinement, and determine the feasibility of integrating the strategies into routine primary healthcare practice. The Modified Delphi process described in the preceding validation phase established expert consensus regarding the clarity, relevance, feasibility, practicality, and applicability of the strategies; however, their effectiveness can only be established through subsequent implementation and empirical evaluation in real world healthcare settings.

## 4 Findings

The findings of this study extend beyond hypertension as a single disease area by demonstrating how climate change adaptation can be integrated into chronic disease management and broader public health planning. Although hypertensive patients were the focus population, the identified challenges including limited awareness of climate related health risks, inadequate preparedness for extreme weather events, inconsistent coping practices, and gaps in climate health communication reflect broader vulnerabilities experienced by individuals living with chronic conditions and other climate sensitive health risks. These findings highlight the need for health systems to move beyond disease specific approaches towards integrated climate resilient planning that addresses shared adaptation needs among vulnerable populations.

The study provided insights into hypertensive patients’ lived experiences of managing their condition under changing weather conditions, including the perceived effects of extreme temperatures, barriers to self-management, existing coping practices, and patient recommendations for strengthening adaptation. These findings informed the development of climate adaptation strategies aimed at enhancing the capacity of hypertensive patients to respond to climate related health challenges in Limpopo Province, South Africa.

The study identified the following key gaps affecting hypertensive patients’ ability to adapt to changing weather conditions:

- Limited knowledge regarding the potential effects of climate change on hypertension and overall health.
- Challenges experienced while living with hypertension under changing weather conditions, particularly during periods of extreme temperatures.
- Reliance on individual coping strategies in the absence of structured climate health guidance.
- The need for improved patient education, communication, and support mechanisms to strengthen climate adaptation.

Based on these findings, seven evidence informed strategies were developed to strengthen adaptive capacity at individual, community, healthcare system, and policy levels. Table 3 illustrates the alignment of these strategies with RAM demonstrating how they address multiple adaptive modes in supporting responses to climate related health stressors among hypertensive patients (15). The physiological-physical mode is addressed through strategies supporting blood pressure management, hydration practices, and adaptation to temperature-related stressors, while the self-concept mode is strengthened through patient education, empowerment, and adaptive coping approaches.

**Table 3:** Mapping of coping strategies to Roy’s adaptation model adaptive modes.

Strategy	Description of strategy	Primary RAM adaptive mode(s)	Explanation of alignment with Roy’s adaptation model
Strategy 1: Climate informed healthcare services	Integration of climate awareness into routine hypertension care, including patient education, Climate resilient facilities, and provider training	Physiological–Physical; Role Function	Addresses physiological responses to climate stressors (e.g., BP instability during heat/cold) while strengthening healthcare providers’ roles as facilitators of adaptive responses
Strategy 2: Selfcare management during extreme heat	Hydration guidance, cooling strategies, heat symptom recognition, and safe outdoor practices	Physiological–Physical	Supports physiological integrity by promoting effective bodily regulation in response to extreme temperatures
Strategy 3: Behavioural and lifestyle interventions	Diet modification, physical activity guidance, stress management, and medication adherence support	Physiological–Physical; Self-Concept	Enhances physical adaptation through lifestyle regulation and supports psychological adaptation by promoting confidence in self-management
Strategy 4: Information coping strategies	Education on climate health risks, BP monitoring, emergency awareness, and peer support	Self-Concept; Interdependence	Strengthens adaptive cognition, self-awareness, and coping confidence while encouraging social support and community engagement
Strategy 5: Patient education and empowerment	Climate health education, self-monitoring, medication management, and emergency response training	Self-Concept; Role Function	Reinforces patients’ self-concept by increasing autonomy and empowers them to actively fulfil their role in managing hypertension
Strategy 6: Climate resilient lifestyle education	Continuous education for healthcare workers and home based carers; community outreach initiatives	Role Function; Interdependence	Enhances the adaptive roles of providers and carers and promotes sustained supportive relationships within communities
Strategy 7: Emergency preparedness strategy	Weather alerts, emergency response planning, medication continuity, and post-event follow up care	Physiological–Physical; Interdependence	Protects physiological stability during climate emergencies and strengthens reliance on support networks during crises

The alignment with RAM further demonstrates that climate adaptation is not solely an individual responsibility but involves interactions between patients, healthcare systems, and social support structures. The physiological-physical mode highlights the importance of addressing environmental stressors, such as extreme temperatures and dehydration, which may also influence individuals living with other chronic conditions, including cardiovascular, respiratory, and renal diseases. The self-concept mode emphasises the role of knowledge, confidence, and empowerment in enabling individuals to make informed decisions regarding their health under changing environmental conditions. The role function and interdependence modes demonstrate the importance of strengthening responsibilities among healthcare providers, community health workers, caregivers, communities, and policymakers through coordinated support systems (16).

The developed strategies operationalise these adaptive modes by strengthening the roles of healthcare providers, home based carers, patients, and community structures in implementing climate responsive hypertension management approaches. In addition, community engagement, social support networks, and emergency preparedness mechanisms support the interdependence required for effective climate adaptation. This theoretical integration demonstrates the applicability of Roy’s Adaptation Model as a guiding framework for climate-sensitive chronic disease management within resource constrained public health settings.

Beyond individual patient adaptation, the developed framework provides a structured mechanism for integrating climate change adaptation into health system planning. The strategies connect patient level adaptive behaviours with healthcare preparedness, multisectoral collaboration, and policy action. Implementation requires collaboration among healthcare services, environmental and meteorological agencies, disaster management structures, local government, and community organisations. For example, climate informed healthcare services and emergency preparedness strategies require the integration of climate information, early warning systems, continuity of care mechanisms, and resource planning into routine healthcare delivery. Similarly, patient education and communication strategies support communities in understanding and responding effectively to climate related health risks.

By transforming patient experiences and coping practices into structured adaptation strategies, this study contributes to the broader climate health agenda by demonstrating how climate change adaptation can be operationalised within public health systems. The findings support a shift from reactive responses to climate related health consequences towards proactive prevention, preparedness, and resilience-building. Although developed within the context of hypertension, the principles underlying the framework including climate informed healthcare delivery, patient empowerment, risk communication, behavioural adaptation, community support, emergency preparedness, and resilient service planning are applicable to wider non-communicable disease programmes and other populations affected by climate related health threats. The developed framework therefore provides a scalable model for strengthening climate resilient healthcare by linking individual adaptive capacity with organisational readiness and policy action. Through evidence informed planning and cross-sectoral collaboration, the framework supports sustainable health system transformation and contributes to SDG 3 and 13.

## 5 Discussion

The developed climate change adaptation strategies provide a structured approach to strengthening hypertension management while addressing the increasing health risks associated with climate variability. Collectively, the strategies address adaptation at the patient, community, healthcare-provider, and health system levels. They align with Sustainable Development Goal (SDG) 3 by promoting access to quality healthcare, strengthening hypertension self-management, and reducing preventable climate related cardiovascular risks. They also contribute to SDG 13 by enhancing adaptive capacity, strengthening climate resilience, and supporting the integration of climate considerations into healthcare planning and service delivery.

Importantly, the findings shift climate responsive hypertension care from a siloed approach to integrated primary care systems planning. Primary healthcare should serve as the central point for linking patient self-management, healthcare provider training, community engagement, climate health information, emergency preparedness, and continuity of treatment. Integrating these components into routine PHC services can enable healthcare providers to identify patients at increased climate related risk, provide timely and consistent education, strengthen self-management, maintain medication and follow up during climate related disruptions, and coordinate referrals and community support. This approach can strengthen communication across levels of care, improve continuity and quality of hypertension management, and enable primary healthcare services to anticipate and respond proactively to climate related risks rather than relying primarily on reactive care.

### 5.1 Strategy 1: climate informed healthcare services

Climate informed healthcare services involve integrating climate change considerations into routine hypertension management through patient education, climate resilient healthcare infrastructure, healthcare provider training, and appropriate health system planning. The primary aim of this strategy is to strengthen the capacity of healthcare services to identify and respond to climate related risks while empowering hypertensive patients with the knowledge and skills required to understand changing weather conditions and adopt appropriate adaptive behaviours.

This strategy was developed in response to findings from the integrated study, particularly limited awareness of the impacts of climate change on hypertension and variations in patients’ experiences of extreme heat and cold. Increasing awareness of hypertension related risks and the influence of climate change represents an important adaptation approach, particularly among vulnerable populations (17). This can be supported through targeted public health communication, patient education, community based interventions, and the integration of climate health considerations into routine primary healthcare services.

At the health system level, policies should support research, surveillance, and interventions addressing the impacts of climate change on people living with hypertension. Integrating climate related considerations into non communicable disease policies can facilitate the development of sustainable and climate responsive healthcare systems (2). Healthcare facilities should also be strengthened to withstand climate related disruptions through appropriate infrastructure, backup power systems, climate-control measures, and emergency continuity plans (18).

Community engagement should complement health system interventions by improving awareness of climate change, hypertension related risks, and locally appropriate adaptation practices. Community based programmes can strengthen participation and collective responses to climate related health challenges (19). Healthcare providers should also promote psychological preparedness and strengthen support networks involving family members, healthcare workers, home based carers, and community organisations, particularly during climate related emergencies (20).

Rather than duplicating patient education across multiple strategies, patient and caregiver education should be integrated as a core component of climate-informed healthcare services and implemented according to individual and environmental needs. This includes education on managing hypertension during extreme heat and cold, appropriate hydration, safe environmental exposure, medication storage, access to cooling or warming facilities, and available community resources (21).

Overall, this strategy establishes the health system foundation for climate responsive hypertension care by integrating climate considerations into policy, infrastructure, workforce preparedness, community engagement, and routine service delivery. It therefore supports both SDG 3 and SDG 13 through the development of sustainable and climate-resilient healthcare services.

### 5.2 Strategy 2: self-management during extreme heat

This strategy focuses specifically on supporting hypertensive patients during periods of extreme heat through practical self-management measures, including hydration, cooling, recognition of heat related symptoms, and safe outdoor practices. Its primary aim is to equip patients with the knowledge and skills required to minimise the health risks associated with increasing temperatures.

The strategy was developed in response to participants’ reported negative experiences during periods of extreme heat. Increasing temperatures may influence cardiovascular function because heat exposure causes peripheral vasodilation, increases cardiovascular workload, and may contribute to fluctuations in blood pressure (22). Extreme heat also increases the risk of dehydration because the body relies on sweating and evaporation to regulate internal temperature. When environmental temperatures become excessively high, thermoregulation may become less effective, increasing vulnerability to heat related complications among individuals with hypertension (23).

Responses to climate related events, including extreme heat, cold, floods, and droughts, are influenced by previous experiences, perceptions, available resources, and access to support systems (24). Therefore, effective adaptation requires both individual self-management and supportive community and healthcare interventions.

During extreme heat, patients should be encouraged to remain indoors or in shaded, well-ventilated environments where possible, take cool showers, minimise outdoor activities during peak temperatures, and use appropriate protective measures when outdoor exposure is unavoidable (25, 26). Light-coloured, loose-fitting clothing and measures such as hats or umbrellas may reduce direct exposure to sunlight (26). Adequate fluid intake should be emphasised to reduce the risk of dehydration and support cardiovascular stability (27).

Healthcare providers should ensure that patients understand warning signs of heat related illness and know when to seek medical assistance. Where available, patients should also be linked with community resources such as cooling centres and local support services (27).

This strategy therefore complements the broader patient education and health system interventions described in Strategies 1 and 5 by providing specific, practical self-management measures for extreme heat. It contributes to SDG 3 by strengthening hypertension self-management and reducing preventable heat related complications, while supporting SDG 13 through enhanced individual and community adaptive capacity.

### 5.3 Strategy 3: Behavioural and lifestyle interventions

This strategy focuses on dietary practices, physical activity, stress management, and medication adherence to promote healthy behaviours and strengthen the capacity of hypertensive patients to manage climate related health challenges. It was developed in response to variations identified in patients’ coping practices and recognises that effective climate adaptation requires the integration of lifestyle modification with clinical management and social support (28).

The ability of hypertensive patients to adapt to climate change is influenced by cognitive, social, and environmental factors, including health literacy, social support, exposure to climate related stressors, and access to appropriate healthcare services (29). Social support can facilitate self-management by providing practical assistance, such as medication reminders, transportation to healthcare appointments, and encouragement to maintain healthy lifestyle practices. Evidence suggests that social support is associated with appropriate dietary practices, physical activity, and treatment adherence among people managing hypertension (30).

Dietary interventions should reinforce established hypertension-management principles while considering climate related conditions. Adequate hydration should be incorporated into dietary counselling, particularly during periods of extreme heat. Physical activity should be tailored to patients’ health status, functional capacity, and environmental conditions, with strenuous outdoor activity avoided during periods of extreme heat or cold (31, 32).

Stress-management interventions should incorporate behavioural counselling, psychological support, and available social networks to assist patients in managing climate related stress and anxiety (33). Medication management should focus on identifying barriers to adherence, providing clear information about prescribed treatment, and reinforcing continuity of treatment during changing environmental conditions (34).

Unlike Strategy 2, which addresses immediate self-management during extreme heat, this strategy focuses on sustained behavioural and lifestyle practices that support long term hypertension management across varying climatic conditions. By strengthening healthy behaviours, self-management capacity, and social support, the strategy contributes to SDG 3 through improved hypertension management and reduced cardiovascular risk and to SDG 13 through strengthened individual adaptive capacity.

### 5.4 Strategy 4: information and coping strategies

This strategy focuses on access to reliable climate health information, blood pressure monitoring, emergency awareness, and peer support to facilitate informed decision making and adaptive behaviours among hypertensive patients (35). It was developed in response to identified gaps in patients’ knowledge regarding climate change, its health implications, and appropriate coping mechanisms during changing weather conditions.

Guided by Roy’s Adaptation Model (RAM), the strategy aims to strengthen patients’ knowledge, confidence, and adaptive capacity to respond to environmental changes (36). Access to accurate and understandable information enables patients to recognise climate related health risks, adopt preventive behaviours, monitor changes in their health status, and seek appropriate care when necessary.

Implementation should therefore include structured communication on the relationship between climate change and hypertension, recognition of climate related symptoms, appropriate self-monitoring, and when to seek medical assistance (37–39). Regular blood pressure monitoring and routine healthcare consultations should be encouraged, particularly for patients experiencing changes in health status during periods of extreme weather.

Peer-support and community based information-sharing mechanisms can further strengthen coping by reducing isolation, facilitating knowledge exchange, and promoting collective adaptation among hypertensive patients (40). In this context, the strategy complements the education components of Strategies 1 and 5 by focusing specifically on the availability, accessibility, and use of information to support decision making.

The strategy contributes to SDG 3 by strengthening health literacy, disease self-management, and early recognition of climate related health risks. It also contributes to SDG 13 by enhancing climate awareness and supporting adaptive responses among vulnerable populations.

### 5.5 Strategy 5: patient education and empowerment

This strategy focuses on strengthening patients’ capacity to participate actively in managing hypertension and responding to climate related health risks. It incorporates self-monitoring, medication management, emergency response knowledge, and patient empowerment within the broader climate health education framework.

The strategy was developed in response to coping practices identified among hypertensive patients and the need to strengthen their ability to manage environmental health risks. Guided by RAM, health is conceptualised as a dynamic state in which individuals continuously adapt to changes within their internal and external environments (41). Hypertension represents an ongoing health experience in which individuals must balance disease management with environmental challenges. Effective adaptation may support the maintenance of health and well-being, whereas inadequate adaptation may increase vulnerability to adverse health outcomes (42).

Patient empowerment should therefore extend beyond the provision of information by developing practical skills for self-management and decision making. Patients should be supported to recognise climate related health risks, monitor their blood pressure where appropriate, understand their prescribed treatment, and identify warning signs requiring professional attention (43). Medication adherence and appropriate storage should be incorporated into this education, particularly where extreme temperatures may affect medication management. Home based carers may provide additional support for patients who require assistance with treatment adherence and continuity of care (44).

Emergency preparedness education should also ensure that patients understand how and when to access healthcare services during climate related events. Guidance should address warning signs requiring urgent medical attention and appropriate actions during emergencies. Hydration and safe physical activity should be addressed within the broader context of individualised hypertension management rather than repeated as separate recommendations (45).

The distinctive contribution of this strategy is therefore the empowerment of patients to apply climate health information in their own daily management of hypertension. Through enhanced self-management capacity and patient participation, the strategy contributes to SDG 3 and strengthens individual and community adaptive capacity in support of SDG 13.

### 5.6 Strategy 6: climate resilient lifestyle education

This strategy focuses on strengthening the capacity of healthcare workers and home based carers to provide consistent, evidence-informed climate health education and support to hypertensive patients. It complements the patient-focused strategies by addressing the capacity of the health workforce to facilitate climate adaptation.

The strategy was developed in response to identified gaps in support, climate health education, and guidance provided by healthcare workers and home based carers (46). Within the framework of RAM, healthcare providers act as facilitators of adaptation by assessing patients’ responses to environmental changes and supporting the development of appropriate coping responses.

According to Onyeneke et al., adaptation involves identifying ineffective coping responses and promoting adaptive behaviours that enhance individuals’ ability to manage environmental stressors (35). Healthcare workers and home based carers should therefore be equipped with the knowledge and skills required to provide climate health education and support. Training may cover climate related lifestyle modification, hypertension management during extreme weather, emergency preparedness, stress management, medication adherence, and appropriate referral pathways (45, 47).

Rather than creating separate education programmes for each patient group, climate health education should be integrated into existing primary healthcare services and delivered through appropriate platforms, including routine consultations, community outreach, household visits, community radio, and other locally accessible communication channels. Such integration can strengthen continuity of care, improve consistency of health messaging, and support community resilience (47).

This strategy contributes to SDG 3 by strengthening health workforce capacity and improving access to quality health information. It supports SDG 13 by embedding climate resilience within healthcare delivery and community based health promotion.

### 5.7 Strategy 7: emergency preparedness strategy

This strategy focuses on strengthening preparedness among hypertensive patients and healthcare services for climate related emergencies through reliable weather alerts, emergency response planning, medication continuity, and post event follow up. It was developed in response to challenges experienced by hypertensive patients during extreme weather events, which may affect physical health, healthcare access, household resources, and psychological wellbeing (48).

Climate related events, including heatwaves, floods, droughts, and cold spells, may disrupt healthcare access and contribute to physiological and psychosocial stress. Within RAM, the physiological-physical mode explains individuals’ physical responses to environmental stimuli, including temperature fluctuations and other climate related stressors. Exposure to extreme heat or cold may result in physiological responses such as dehydration, vasodilation, vasoconstriction, elevated blood pressure, and increased cardiovascular strain (35).

Preparedness should begin with access to reliable weather information through official alerts, healthcare communication channels, and trusted media sources. Early warning information enables patients to modify their behaviour and take preventive measures before exposure to hazardous conditions. During extreme heat, patients may be at increased risk of dehydration, electrolyte imbalance associated with excessive sweating, heat stress, heat exhaustion, and heatstroke (49). Conversely, extreme cold may contribute to vasoconstriction, increased blood pressure, and cardiovascular complications, including myocardial infarction and stroke. Cold conditions may also affect indoor air quality through certain heating practices, potentially contributing to respiratory irritation and additional cardiovascular stress (50).

At the health system level, emergency response protocols should include contingency arrangements for maintaining continuity of hypertension care, ensuring access to essential medications and medical supplies, and facilitating timely healthcare support during climate related disruptions (51). At the patient level, emergency preparedness plans may include an emergency supply of essential medications, updated medical information, emergency contact details, knowledge of evacuation routes, and information on available healthcare services (52).

Post event care should form an integral component of emergency preparedness. Structured follow-up should be used to assess patients’ health status, identify emerging complications, and provide appropriate clinical support. Where required, psychological support should be incorporated into post-disaster care to address stress and other psychosocial consequences associated with extreme weather events (53).

This strategy provides the emergency response component of the overall adaptation framework and therefore complements, rather than duplicates, the preventive and educational strategies described above. It contributes to SDG 3 by strengthening continuity of care and reducing preventable hypertension related complications during emergencies, while supporting SDG 13 through enhanced climate adaptation capacity and resilient health system preparedness (Table 4).

**Table 4:** Practical roles of the frameworks.

Framework	Practical role in the study
Roy adaptation model (RAM)	Explains how people with hypertension adapt to climate related stressors such as heat, changing weather patterns, food insecurity, and disruptions to healthcare. It helps identify patients’ adaptive needs and guides patient-centred interventions.
Thompson strategy development framework	Provides the process for translating evidence and identified needs into practical strategies. It supports prioritising problems, developing appropriate interventions, and structuring strategies for climate adaptive hypertension care.
Measurement for change (M4C)	Provides the systems level perspective. It helps ensure that strategies are integrated across patients, healthcare providers, facilities, communities, and public health structures, while emphasising measurement, collaboration, learning, sustainability, and continuous improvement.

Development Framework, and Measurement for Change (M4C), although they operate at different levels. RAM focuses on how patients adapt to environmental and health related stressors, while Thompson provides a structured process for developing evidence informed strategies. M4C extends these principles to the health system level, emphasising integration, collaboration, measurement, learning, and sustainability. For resilience planning, the key reflection is that effective climate adaptation cannot rely on patient coping alone. Patient level adaptation must be supported by responsive healthcare providers, appropriate resources and infrastructure, climate information, coordinated services, and continuous monitoring and learning. Together, the frameworks support a shift from individual coping to integrated, system enabled climate resilience, strengthening sustainable and climate responsive hypertension care within primary healthcare.

## 6 Conclusion

Climate change represents an increasing global public health threat with significant implications for hypertension prevention, management, and long term outcomes. These challenges are intensified by health system limitations, socioeconomic vulnerabilities, and unequal access to healthcare resources, particularly in resource constrained settings where extreme weather events can disrupt healthcare delivery and continuity of chronic disease management. This study addresses an important knowledge gap by developing seven evidence informed climate adaptation strategies for hypertensive patients based on empirical evidence from their lived experiences and coping practices. Grounded in RAM, the framework extends beyond individual behaviour change by providing a structured approach for integrating patient education, self-management support, climate responsive healthcare services, emergency preparedness, and community engagement into routine hypertension care. In doing so, it demonstrates how patient generated evidence can be translated into practical strategies that strengthen both individual adaptive capacity and health system responsiveness to climate related health risks.

Importantly, the framework offers a practical process for integrating climate adaptation into chronic disease management and public health planning. Rather than addressing hypertension as an isolated clinical condition, it promotes coordinated action among healthcare providers, policymakers, environmental agencies, disaster management structures, and communities. This systems oriented approach provides a transferable model for incorporating climate considerations into the prevention and management of other climate-sensitive non communicable diseases, particularly within resource constrained settings. The framework therefore contributes to broader efforts to strengthen climate resilient health systems by supporting proactive planning, cross sectoral collaboration, patient centred care, and equitable access to health services. Its implementation has the potential to advance SDG 3 through improved chronic disease management and SDG 13 by strengthening adaptive capacity and resilience within healthcare systems and communities.

Future research should evaluate the implementation, feasibility, and effectiveness of the proposed framework across diverse healthcare settings and examine how digital health innovations, including telemedicine, mobile health applications, climate health information systems, and early warning systems, can further strengthen climate responsive chronic disease management and improve health outcomes among vulnerable populations.

## 7 Study limitations

This study has several limitations. First, the findings are context-specific to selected districts of Limpopo Province and should be generalised cautiously to other regions with different climatic, socioeconomic, cultural, and healthcare-system contexts. Second, the cross-sectional quantitative data relied largely on self-reported experiences and therefore cannot establish causal or long-term relationships between climate-related exposures, coping strategies, and hypertension outcomes. Third, the modified Delphi validation reflected expert consensus and did not assess the effectiveness of the proposed strategies in routine primary healthcare settings. Finally, although the framework integrates empirical evidence, the Roy Adaptation Model, the Thompson Strategy Development Framework, and a systems-oriented approach, its implementation feasibility, scalability, sustainability, cost-effectiveness, and impact on blood pressure control, patient self-management, and climate-resilient primary healthcare remain untested. Future research should therefore use longitudinal and implementation-focused designs, objective clinical and environmental data, broader stakeholder participation, and multi-site validation to evaluate and refine the framework.

## Data Availability

The raw data supporting the conclusions of this article will be made available by the authors, without undue reservation.
